# Book review: *Re-imagining vector control: A modern perspective on forgotten giants*

**DOI:** 10.5281/zenodo.14850486

**Published:** 2025-02-11

**Authors:** Bart G.J. Knols

**Affiliations:** 1 Editor, MalariaWorld Journal

## Abstract

Imagine, for a second, how pioneers in malaria and other vector-borne diseases would look at the current state of affairs in view of the stagnating progress in reducing the global impact of these diseases. In his book, Lluberas takes readers on a quick journey through history and reimagines how these forgotten giants would adapt to the 21st-century battle against vector-borne diseases. The life and work of thirteen pioneers (several of them still alive) is highlighted and culminates in a synthesis that should guide or perhaps even re-shape current and future efforts to combat vector-borne diseases.

Imagine that nobody believes in what you are doing, or in what you are proposing to do. That your ideas are being laughed at or completely ignored. But that against all odds, you persist, remain driven, passionate, and then, ultimately, you succeed. Some of the earliest pioneers in vector-borne disease research faced exactly those circumstances. Yet, we view them today as heroes, out-of-the-box thinkers, and as people that had guts. Regretfully, many of these giants and their seminal works have been forgotten.

In today's world this pioneering is hardly imaginable, now that we have organised science very differently. Before creative thinking and revolutionary ideas can be executed, most will be shot down by review committees or simply be refused by PhD supervisors. Past pioneers, like Dr. Fred Soper (covered by a chapter in the book), proposed elimination of an invasive African malaria vector from north-east Brazil, and was ridiculed. This had never been done before and seemed an outrageous idea. But he did it, against all odds. And was successful.

This is where Lluberas’ book brings important value. In his own words: “Ultimately, *Reimagining Vector Control* is more than a historical retrospective; it is a call to rethink and modernise vector control by drawing inspiration from those who paved the way. By resurrecting the lost strategies and scientific philosophies of these pioneers, the book challenges readers to envision a world where their forgotten lessons fuel innovative, effective, and sustainable disease control efforts for the future”.

The book describes the work and impact of thirteen pioneers. I must admit, that two out of these thirteen were unknown to me, and for someone interested in both historical and contemporary research and control of mosquito-borne diseases with more than 35 years of experience, this is surprising. Several of the giants selected would be expected by many to feature prominently, like Sir Ronald Ross, Sir William Gorgas, Dr. Fred Soper, or Dr. Israel Kliger. Each of these pioneers had a massive impact on the field of tropical medicine and mosquito-borne disease control. As the story moves into the second half of the last century, Lluberas then selects giants that beyond doubt earned their credentials in the scientific research arena but arguably have not changed our field as dramatically as the ‘early’ pioneers. I find it difficult to equate the merits of eliminating malaria from Palestine (Kligler) with the development of a gene drive system (James) that has never been used in the real world and merely ‘offers hope’, to use Lluberas’ own words. In the latter case, there are many ‘pioneers’, dead or alive, that would pass the criterion of having ‘offered’ hope. Africa’s Wenceslaus Kilama or Botha de Meillon, UK’s Chris Curtis or Sir Brian Greenwood, or USA’s Walter Reed, to name but a few, could easily have featured as giants, based on their massive contributions that actually reached much further than offering hope. So the exact criteria on which Lluberas based his choice for ‘giants’ remains somewhat vague, and he admits that this wasn’t an easy task. For the giants in the book that are alive today, it would have been nice to incorporate their views and opinions, but the book is not showing any signs of direct interaction between the author and these scientists, which is a pity. In the opening chapters of the book, Lluberas wonders how these giants would have coped with today’s challenges of climate change or insecticide resistance, and the ones alive today could have provided interesting input regarding these issues.

Criticism aside, the book does provide a great start for beginners to know what their predecessors were like and what they achieved. But for veterans in mosquito control it serves equally well as a reminder that each and every one of us could change our field if we had the same spirit, drive and passion (and perhaps one day turn into a giant also, who knows).

Using his initial four giants, Ross, Gorgas, Soper, and Kligler as excellent examples, Lluberas describes what could be their likely views on how we do mosquito control today. Ross would likely exploit the merits of computer-aided modelling and exploit the predictive power thereof to the fullest to improve vector management campaigns. Gorgas Soper and Kligler would marvel at AI-guided surveillance with drones and would immediately reap the benefits of these technological developments in their military style campaigns. For them to see a drone disappearing on the horizon to look for standing water on its own, apply a larvicide on it and return to base would be sheer science fiction. They would make full use of satellite imagery, GIS and GPS tools to make their campaigns much more (cost)efficient. And if they were alive, they would probably push very hard for all of these technological developments to be incorporated in current campaigns - which, it can be argued, we’re not doing enough. But at the same time they would be astonished to see how little of their proven and highly successful approaches remain in use today, notably environmental management, large-scale larval source management, and intense and prolonged community engagement. No doubt, they would all ask us some pretty pertinent questions that we would find hard to answer. And again, this is where Lluberas’s book brings good value. It makes us aware of our current shortcomings and indeed makes us wonder why we seem to have ‘lost’ some of these proven tactics, and perhaps above all, our will and determination to win the battle against mosquito-borne diseases.

The striking work of Gabaldón in Venezuela, who led a programme that back in the 1940s freed an area larger than the size of forty African countries today of malaria serves as another example of what could be achieved if we’d change our commodity-driven approach to a rigorous, integrated, and strategy-driven approach. Daring to ‘think big’ was not a problem for these giants, and they’d be pointing fingers at us for having lost audacity.

Of the many masterminds behind the first Global Malaria Eradication Programme (which ran between 1955-1969), Paul Russell was instrumental in shaping this gigantic task that by many today is described as a failure, yet forgetting that about a quarter of the world’s population today lives without the risk of malaria as a result of this campaign.

The work of Andy Spielman, who moved beyond the indiscriminate use of pesticides through a better understanding of the behaviour and ecology of mosquitoes, partially influenced by Rachel Carson’s *Silent Spring*, published in 1962, was gigantic and influential and both have their own chapter in Lluberas’ book.

Chapters describing the work of today’s giants (Anthony James, Laura Harrington, Maria Sallum Rose Leke, and Carolina Cunha) follow, and these are all worth reading though not as compelling as the chapters about the early giants.

Lluberas ends his book with a clear message, making reference to the early pioneers: “If these visionaries were alive today, they would not merely adopt and adapt to modern technologies and challenges; they would lead the way in redefining what is possible in global malaria control. Their leadership would underscore that malaria is not an unsolvable problem but a challenge that demands innovation, partnership, action, and an unwavering commitment to human health”.

Indeed, a very clear message for us all.

